# Supplementary material for: EnzML: multi-label prediction of enzyme classes using InterPro signatures
Source: BMC Bioinformatics. 2012 Apr 25;13:61. doi: 10.1186/1471-2105-13-61 (PMC3483700; doi:10.1186/1471-2105-13-61)
Supplement: Addtional file 5 — The Java code to format the data files, evaluate and predict. The file enzml_java_code.tar.gz contains the Java code used to format database data to ARFF and XML formats, to execute cross and train-test (jackknife) evaluations and to record evaluation results to database. More information is included in the readme.txt file and the Javadoc files. The code can be used with a MySQL database. To use a different database software, other JDBC drivers might be required. [file 1471-2105-13-61-S5.gz › java_code/utils/doc/uk/ac/ed/inf/utils/tests/Data.html]

Data


---


|  |  |  |
| --- | --- | --- |
| **Overview** \| **Package** \| **Class** \| **Use** \| **Tree** \| **Deprecated** \| **Index** \| **Help** | |  |
| **PREV CLASS**   **NEXT CLASS** | **FRAMES**    **NO FRAMES**     **All Classes** |  |
| SUMMARY: NESTED \| FIELD \| CONSTR \| METHOD | DETAIL: FIELD \| CONSTR \| METHOD |  |


---


## uk.ac.ed.inf.utils.tests Class Data

```
java.lang.Object
  junit.framework.Assert
      junit.framework.TestCase
          uk.ac.ed.inf.utils.tests.Data
```

**All Implemented Interfaces:**: junit.framework.Test

---

``` public class Data extends junit.framework.TestCase ```

Class

**Version:**
:   3 Jun 2008

**Author:**
:   Luna De Ferrari luna.deferrari-at-ed.ac.uk

---

| **Field Summary** | |
| --- | --- |
| `static java.lang.String` | `KEY1` |
| `static java.lang.String` | `KEY2` |
| `static java.lang.String` | `KEY3` |
| `static java.lang.String` | `KEYA` |
| `static java.lang.String` | `VAL1` |
| `static java.lang.String` | `VAL2` |
| `static java.lang.String` | `VAL3` |
| `static java.lang.String` | `VALA` |
| `static java.lang.String` | `VALB` |


**Constructor Summary**

`Data()`


| **Method Summary** | |
| --- | --- |
| `static java.util.Vector<java.lang.String>` | `keys()` |
| `static java.util.Vector<java.lang.String>` | `keys2()` |
| `static java.util.Vector<java.lang.String>` | `values()` |
| `static java.util.Vector<java.lang.String>` | `values2()` |

**Methods inherited from class junit.framework.TestCase**

`countTestCases, getName, run, run, runBare, setName, toString`

**Methods inherited from class junit.framework.Assert**

`assertEquals, assertEquals, assertEquals, assertEquals, assertEquals, assertEquals, assertEquals, assertEquals, assertEquals, assertEquals, assertEquals, assertEquals, assertEquals, assertEquals, assertEquals, assertEquals, assertEquals, assertEquals, assertEquals, assertEquals, assertFalse, assertFalse, assertNotNull, assertNotNull, assertNotSame, assertNotSame, assertNull, assertNull, assertSame, assertSame, assertTrue, assertTrue, fail, fail, failNotEquals, failNotSame, failSame, format`

**Methods inherited from class java.lang.Object**

`equals, getClass, hashCode, notify, notifyAll, wait, wait, wait`

**Field Detail**

### KEY1

```
public static final java.lang.String KEY1
```

**See Also:**: Constant Field Values

---


### KEY2

```
public static final java.lang.String KEY2
```

**See Also:**: Constant Field Values

---


### KEY3

```
public static final java.lang.String KEY3
```

**See Also:**: Constant Field Values

---


### KEYA

```
public static final java.lang.String KEYA
```

**See Also:**: Constant Field Values

---


### VAL1

```
public static final java.lang.String VAL1
```

**See Also:**: Constant Field Values

---


### VAL2

```
public static final java.lang.String VAL2
```

**See Also:**: Constant Field Values

---


### VAL3

```
public static final java.lang.String VAL3
```

**See Also:**: Constant Field Values

---


### VALA

```
public static final java.lang.String VALA
```

**See Also:**: Constant Field Values

---


### VALB

```
public static final java.lang.String VALB
```

**See Also:**: Constant Field Values


**Constructor Detail**

### Data

```
public Data()
```


**Method Detail**

### keys

```
public static java.util.Vector<java.lang.String> keys()
```

---


### keys2

```
public static java.util.Vector<java.lang.String> keys2()
```

---


### values

```
public static java.util.Vector<java.lang.String> values()
```

---


### values2

```
public static java.util.Vector<java.lang.String> values2()
```


---


|  |  |  |
| --- | --- | --- |
| **Overview** \| **Package** \| **Class** \| **Use** \| **Tree** \| **Deprecated** \| **Index** \| **Help** | |  |
| **PREV CLASS**   **NEXT CLASS** | **FRAMES**    **NO FRAMES**     **All Classes** |  |
| SUMMARY: NESTED \| FIELD \| CONSTR \| METHOD | DETAIL: FIELD \| CONSTR \| METHOD |  |


---
